# Comparative Effectiveness of Injection Therapies for Hemiplegic Shoulder Pain in Stroke: A Systematic Review and Network Meta-Analysis

**DOI:** 10.3390/ph14080788

**Published:** 2021-08-10

**Authors:** Yi-Hsiang Chiu, Ke-Vin Chang, Wei-Ting Wu, Po-Cheng Hsu, Levent Özçakar

**Affiliations:** 1 Department of Physical Medicine and Rehabilitation, National Taiwan University College of Medicine, Taipei 10048, Taiwan; Chiu19910703@gmail.com; 2 Department of Physical Medicine and Rehabilitation, National Taiwan University Hospital, Bei-Hu Branch, Taipei 10845, Taiwan; wwtaustin@yahoo.com.tw (W.-T.W.); myronrbman@gmail.com (P.-C.H.); 3 Center for Regional Anesthesia and Pain Medicine, Wang-Fang Hospital, Taipei Medical University, Taipei 11600, Taiwan; 4 Department of Physical and Rehabilitation Medicine, Hacettepe University Medical School, 06100 Ankara, Turkey; lozcakar@yahoo.com

**Keywords:** corticosteroid, hemiplegic shoulder, hyaluronic acid, injection, rehabilitation

## Abstract

Hemiplegic shoulder pain (HSP) hampers post-stroke functional recovery and is not well managed with conservative treatments. This systematic review aimed to examine the various injection therapies for HSP and investigate their effectiveness at different time points. The protocol of this meta-analysis was registered on INPLASY with a registration number of INPLASY202180010. PubMed, EMBASE, and Scopus were searched from their inception to 4 August 2021 for the clinical studies investigating comparative effectiveness of different injection regimens for treating hemiplegic shoulder pain in patients with stroke. The primary outcome was the weighted mean difference (WMD) on the visual analog scale (VAS) of pain reduction in the fourth-week and between the fourth and twenty-fourth weeks. Ranking probabilities of the WMD for each treatment were obtained using simulations. Seventeen studies with 595 participants were included. The network meta-analysis showed that at the fourth-week, intra-muscular botulinum toxin (BoNT) injections and suprascapular nerve blocks (SSNB) were superior to a placebo, with WMDs of 1.55 (95% CI, 0.09 to 3.01) and 1.44 (95% CI, 0.07 to 2.80), respectively. SSNB possessed the highest probability (53.3%) and appeared to be the best treatment in the fourth-week, followed by intra-muscular BoNT injections (42.6%). Intramuscular BoNT injections were better than the placebo, with a WMD of 1.57 (95% CI, 0.30 to 2.84) between the 4th and 24th weeks. Intramuscular BoNT injections had the highest probability (79.8%) as the best treatment between the 4th and 24th weeks. SSNB was likely to rank first in relieving HSP at the fourth post-treatment week, whereas intra-muscular BoNT injections had the highest probability to achieve the best treatment effectiveness in the post-injection period between the fourth and twenty-fourth weeks. However, as some of the included studies used a non-randomized controlled design, more randomized controlled trials are needed in the future to validate and better understand the short- and long-term efficacy of different injection therapies for management of HSP.

## 1. Introduction

Hemiplegic shoulder pain (HSP) is one of the most debilitating complications after stroke [1]. Its reported incidence varies from 30% to 72% at one-year follow-up across different studies [2,3,4]. HSP is mostly graded as ranging from moderate to severe intensity [4] and rarely resolves spontaneously [3]. Stroke patients with poor upper extremity function have an increased risk of HSP [5]. Various theories have been proposed for the development of HSP, including deficiency in pain adaption [6], central sensitization to normal or subthreshold sensory stimuli [7], and impaired neuromuscular control of the scapula [8]. Spasticity over the hemiplegic limbs, shoulder subluxation, concomitant rotator cuff pathology, and prolonged immobilization of the affected limbs are also reported to be associated with HSP [9]. Without adequate management, HSP further worsens the function of the upper extremities and can prolong the hospital stay [2].

Shoulder slings, passive range of motion exercises, analgesics, and electrical stimulation have been commonly applied for treating HSP, although their effects are usually limited [10]. In recent years, various injection therapies have been proposed in its management. For instance, suprascapular nerve block (SSNB) using local anesthetics can be performed to decrease nociception from the glenohumeral joint [11]. Intramuscular botulinum toxin (BoNT) injections are also effective in reducing spasticity of the hemiplegic limbs and the associated pain [12]. On the other hand, BoNT injections are beneficial for decreasing chronic shoulder pain, possibly through inhibition of the release of pain mediators [13]. Corticosteroid injections have long been used to treat painful shoulders owing to their anti-inflammatory potential, while intra-articular hyaluronic acid (HA) injections might prevent adhesions and reduce synovitis inside the glenohumeral joint [14]. Until now, no meta-analysis has been conducted to investigate which injection regimen is the best for the management of HSP. Therefore, we aimed to perform a systematic review of the evidence regarding injection therapies to treat HSP with a network meta-analysis, comparing their effectiveness at different time points following interventions.

## 2. Methods

The current network meta-analysis was conducted according to the Preferred Reporting Items for Systematic Reviews and Meta-Analyses extension guidelines for network meta-analysis (PRISMA-NMA) [15]. All supporting data are available within the article and its online supplementary files. The protocol of this meta-analysis was registered on INPLASY (International Platform of Registered Systematic Review and Meta-analysis Protocols) with a registration number of INPLASY202180010 (https://inplasy.com/inplasy-2021-8-0010/ (accessed on 3 August 2021)).

### 2.1. Search Strategy

We conducted a systemic review of PubMed, EMBASE and Scopus from the earliest records to 4 August 2021. Manual searches for eligible articles from the reference list of review articles and meta-analyses were also applied. The following PICO (i.e., Population/Patient, Intervention, Comparison, Outcome) question guided the search strategy: “In patients with stroke, which kind of injection therapy, in comparison to other injection or placebo treatments, has a better effect in relieving hemiplegic shoulder pain?”. The following keywords were used: “stroke”, “cerebrovascular disease”, “cerebral infarction”, “intracerebral hemorrhage”, “hemiplegia”, “hemiparesis”, “injection”, “nerve block”, “corticosteroid”, “botulinum toxin”, “hyaluronic acid”, “shoulder”, “upper limb”, “pain”, and “painful”. The databases were investigated based on the following algorithm: (stroke OR cerebrovascular disease OR cerebral infarction OR intracerebral hemorrhage OR hemiplegia OR hemiparesis) and (injection OR nerve block OR corticosteroid OR botulinum toxin OR hyaluronic acid) and (shoulder OR upper limb) and (pain OR painful). A detailed search strategy is provided in the supplement (Supplemental Method). No language restriction was imposed on the literature search.

### 2.2. Inclusion and Exclusion Criteria

In the network meta-analysis, we included clinical studies employing any type of injection therapy against HSP in patients who had a stroke, which could be randomized controlled trials (RCTs), quasi-experimental trials and cohort observational studies. No limitations were imposed on the stroke type and chronicity or on the concomitant therapy after the injections. To constitute a network meta-analysis for comparison, the enrolled studies were required to have at least two arms of injection therapies if they did not include a placebo group. Studies were excluded if they: (1) investigated shoulder pain on non-stroke patients; (2) did not employ injection therapies to treat HSP; (3) used needling without administrating regimens; (4) injected autologous blood-derived products (for the concern of significant variations in plasma components among different individuals); and (5) lacked serial measurements of shoulder pain. In cases of overlapping patients in multiple studies, only the latest publication was included in the analysis.

### 2.3. Data Extraction, Quality Assessment, and Evaluation of Inconsistency

Two authors independently assessed the reports for eligibility. After any duplicates were removed, all the titles, abstracts, and full texts of the eligible articles were screened. The reasons for exclusion were recorded. The data were reviewed for consistency between the two authors, and any discordance was resolved by discussion with the corresponding author. The following items, including author, year, trial design, allocation, inclusion criteria, stroke characteristics, case number, average age, details of the intervention, length of follow-up, and relevant outcomes, were extracted from the enrolled studies. The study quality was evaluated using the Cochrane Collaboration tool for assessing the risk of bias [16], which reviews the randomization/allocation of the participants, blindness of interventions for patients and outcome assessors, and completeness of data reporting. The assessment was based on the version 2 of Cochrane risk-of-bias tool, and the items were graded as having a high, low, or unclear risk of bias [16].

The statistical method used for the evaluation of the agreement of direct and indirect evidence was the loop inconsistency model [17]. Inconsistency was able to be identified when the disagreement existed between various origins of evidence within a closed loop. In each loop, the inconsistency factor was calculated for the difference in the pooled effect size between the direct and indirect comparisons, which was shown on the inconsistency plot. The 95% CI of the inconsistency factor was employed to determine the existence of inconsistency in certain loops [18].

### 2.4. Outcomes

The primary outcome was the weight mean difference (WMD) on the visual analog scale (VAS) of pain reduction in the fourth-week and between the 4th and 24th weeks following the intervention. The WMD of VAS reduction in the fourth week were available in nearly all the trials investigating injection therapies for post-stroke hemiplegic shoulder pain. Furthermore, the 24th weeks were the longest follow-up period available in the eligible studies. Our analysis was based on the measurements of pain intensity on a 10-cm VAS. If a 100-mm VAS was used in the included studies, we would standardize the value in accordance to a 10-cm VAS. In case a numerical rating scale (0–10) was used, the value would be directly employed for a pooled analysis as a surrogate measurement on a 10-cm VAS. Our method of standardization was similar to a recent systematic review, which also reported that the minimal clinically important difference of VAS values ranged from 0.8 to 4 cm [19]. We extracted the VAS at rest for the meta-analysis for those trials that included assessments during different physical conditions. If the value mentioned above was not available, the priority of our choices was the VAS at night first followed by the worst VAS during the day and the VAS during motion. Due to decreased physical activities at night, the pain intensity measured would be similar to the value obtained at rest. However, the worst VAS was mostly reported during shoulder motion but less frequently at rest. In case the authors did not specify at which condition the VAS was measured, we would suppose that the VAS was acquired at rest. The mean and standard deviation were estimated by using the quantile estimation approach proposed by McGrath et al. [20] if the retrieved articles only reported the medians and interquartile range.

### 2.5. Statistical Analysis

Considering variations of disease severity and therapeutic regimens in different studies, we employed a random effect model in the pairwise meta-analysis [21]. Heterogeneity of direct comparisons among the enrolled trials was analyzed and shown using the Cochrane Q tests and I^2^ statistic, respectively [22]. Significant heterogeneity was assumed when the I^2^ value was > 50% [22].

In the network meta-analysis, a mixed treatment comparison with a generalized linear mixed model was used to compute the direct and indirect comparisons [23]. The strength of the network meta-analysis is shown on its capability of indirectly estimating the differences between treatment A and B through calculation from their comparisons with treatment C. The geometry of the treatment network was represented by the network plot [18]. Two chains with different initial values were input simultaneously for the assessment of convergence and the consistency of the model was evaluated through comparing direct and indirect estimates in each triangular loop [24]. Rank probabilities of effectiveness in pain relief of each treatment were obtained using simulations and demonstrated by employing the surface under the cumulative ranking area (SUCRA) and ranking probability curves. The value of SUCRA ranged between 0 and 1 and the intervention with a higher SUCRA value indicated better efficacy. The SUCRA was indicative of the percentage of the mean rank of each treatment in relation to a theoretical therapy that was assumed to be the best effect [18,25]. A higher odd in SUCRA denoted superior ranking [26]. The potential existence of publication bias was examined by inspecting the distribution pattern of all study effects on the funnel plot as well as the *p*-values from Egger’s regression test [27]. Furthermore, a sensitivity analysis was performed by excluding the non-RCTs. All the analyses were conducted using the statistical software package Stata (StataCorp. 2015. Stata Statistical Software: Release 14. StataCorp LP, College Station, TX, USA), and statistical significance was set at a *p*-value of <0.05.

## 3. Results

### 3.1. Study Selection and Characteristics

**Figure 1:**
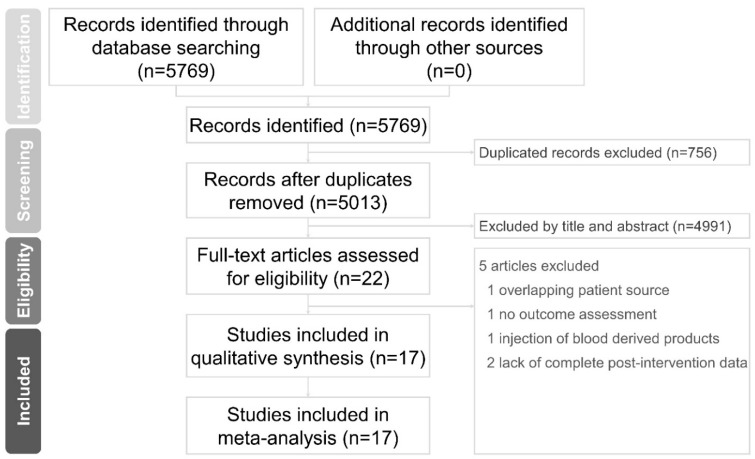
Flow chart of the study selection process.

We identified 5769 citations in the literature search from the electronic databases, PubMed, EMBASE, and Scopus. No additional articles were extracted manually from any of the reference lists for the analysis. A total of 756 articles were removed due to duplication. We later screened the titles and abstracts of the remaining studies and retained 22 articles for full-text assessment. One study [28] was excluded due to an overlapping patient source, one study protocol [29] was excluded due to no outcome assessment, one study [30] was excluded due to use of autologous blood-derived products and two studies [31,32] were excluded due to a lack of complete post-intervention data. The final meta-analysis included 17 articles (Figure 1) comprising 595 participants. Average age, stroke duration, and a summary of the retrieved trials are listed in Table 1.

In the 17 enrolled articles [11,12,14,33,34,35,36,37,38,39,40,41,42,43,44,45,46], five trials compared intra-muscular BoNT injections with a placebo [12,35,36,37,38], one trial compared intramuscular BoNT injections with intra-articular corticosteroid injections [40], one trial compared intra-muscular BoNT injections with SSNB [39], one trial compared intra-bursal BoNT injections with intra-bursal corticosteroid injections [34], three trials compared intra-articular/bursal corticosteroid injections with a placebo [43,44,45], one trial compared SSNB with intra-articular corticosteroid injections [46], three trials compared SSNB with a placebo [11,41,42], one trial compared HA injections with a placebo [14], and one trial compared HA injections with intra-articular corticosteroid injections [33]. Most of the included studies were double- or triple-blinded RCTs [12,14,35,36,37,38,39,40,41,42,44,46], except one single-blinded RCT [33], one unblinded RCT [43], one quasi-experimental study [11] and one retrospective cohort study [34]. Most of the included studies reported the VAS both in the fourth week and between the 4th and 24th weeks. Assessment of pain was not available at the fourth week in three studies [35,40,45], and between the 4th and 24th weeks in three studies [38,42,43]. Injection guidance were based on ultrasonography in seven studies [11,14,33,34,39,42,44], electromyography in three studies [12,37,38], fluoroscopy in one study [46] and landmark in six studies [35,36,40,41,43,45]. The details of the interventions are listed in Table 2.

### 3.2. Assessment of the Study Quality

Figure S1 summarizes the risk of bias assessment for the included studies, and Figure S2 represents the risk of bias graph. The domain that failed the most was in Item 3 (blinding of participants and personnel) followed by Item 4 (blinding of outcome assessment) as some of the studies were not RCTs. Another commonly failed item was in Item 2 (allocation concealment) as some of the studies did not mention whether the research investigators concealed the allocation sequence during the trials.

### 3.3. Assessment of the Inconsistency between the Direct and Indirect Evidence

Regarding the comparison of WMD for VAS reduction at the fourth week, the test of inconsistency from the loop inconsistency model (Figure S3A) did not show any evidence of significant inconsistencies between the direct and indirect comparisons. However, the test of inconsistency from the same model (Figure S3B) revealed significant inconsistencies between direct and indirect comparisons over the loop of placebo-steroid-HA, with an inconsistency factor of 2.66 (95% CI, 0.61–4.70) in terms of the comparison of WMD for VAS reduction between the 4th and 24th weeks.

### 3.4. Comparison of WMD for VAS Reduction (Fourth-Week)

**Figure 2:**
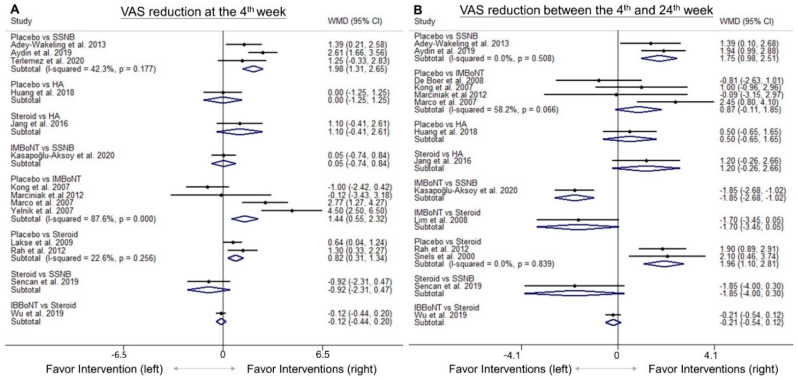
Forrest plots for the available direct comparisons between pairs of treatments among the included studies regarding the effects of VAS reduction (**A**) at the 4th week and (**B**) between the 4th and 24th weeks after interventions. BoNT, botulinum toxin; HA, hyaluronic acid; IB, intra-bursal; IM, intra-muscular; SSNB, suprascapular nerve block; VAS, visual analogue scale; WMD, weight mean difference.

**Figure 3:**
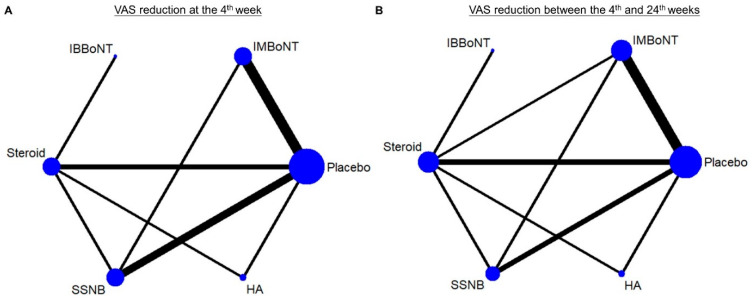
Network plots of injection therapies in terms of the VAS reduction (**A**) at the 4th week and (**B**) between the 4th and 24th weeks. The nodes indicate the types of injections being compared and the edges indicate available direct comparisons between pairs of treatments. BoNT, botulinum toxin; HA, hyaluronic acid; IB, intra-bursal; IM, intra-muscular; SSNB, suprascapular nerve block; VAS, visual analogue scale.

**Figure 4:**
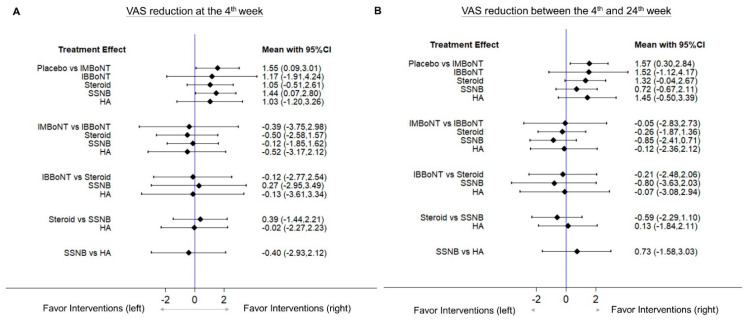
Forest plots of the network estimates derived from all the direct and indirect pairwise comparisons of the treatment effects in terms of VAS reduction (**A**) at the 4th week and (**B**) between the 4th and 24th weeks after interventions. BoNT, botulinum toxin; HA, hyaluronic acid; IB, intra-bursal; IM, intra-muscular; SSNB, suprascapular nerve block; VAS, visual analogue scale.

**Figure 5:**
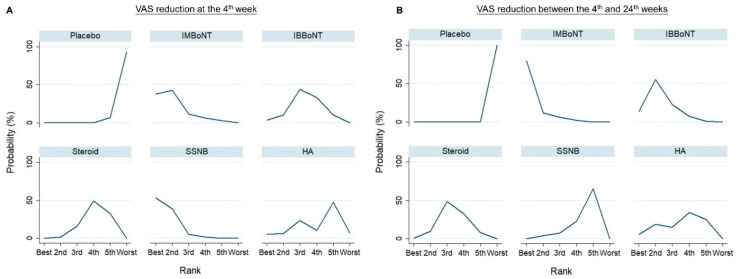
Ranking probabilities for different injection therapies in terms of VAS reduction (**A**) at the 4th week and (**B**) between the 4th and 24th weeks. The plot demonstrates the probabilities of being the best, second best, third best, fourth best, fifth best, and worst among the six injection treatments. BoNT, botulinum toxin; HA, hyaluronic acid; IB, intra-bursal; IM, intra-muscular; SSNB, suprascapular nerve block; VAS, visual analogue scale.

A forest plot of pairwise meta-analysis for the WMDs between different injection therapies is presented in Figure 2A. For the network meta-analysis, the network graph is shown in Figure 3A, disclosing the geometry of the treatment network. The forest plot of network comparisons is presented in Figure 4A. The league tables for the pairwise and network meta-analyses are summarized in Table S1. The rank probability results and the value of SUCRA are presented in Figure 5A and Table 3, respectively.

In the pairwise meta-analysis (Figure 2A), SSNB, intramuscular BoNT injections, and intra-articular/bursal corticosteroid injections were significantly more effective than the placebo, with WMDs of 1.98 (95% CI, 1.31 to 2.65), 1.44 (95% CI, 0.55 to 2.32), and 0.82 (95% CI, 0.31 to 1.34), respectively. There were no significant differences in the following comparisons: intra-articular HA vs. placebo or intra-articular/bursal corticosteroids, SSNB vs. intra-muscular BoNT, or intra-articular/bursal corticosteroids and intra-bursal BoNT vs. intra-articular/bursal corticosteroids.

In the network meta-analysis (Figure 4A), intra-muscular BoNT injections and SSNB were superior to the placebo, with WMDs of 1.55 (95% CI, 0.09 to 3.01) and 1.44 (95% CI, 0.07 to 2.80), respectively. No significant differences were identified regarding the other comparison pairs.

In terms of the fourth-week outcome, the simulation of rank probabilities (Figure 5A) revealed that SSNB possessed the highest probability (53.3%) as the best treatment, whereas intra-muscular BoNT injections had the highest probability (42.6%) as the second-best therapy. Furthermore, intra-bursal BoNT, intra-articular/bursal corticosteroids, and intra-articular HA injections were likely to be the third-, fourth- and fifth-best treatments, with probabilities of 43.6%, 49.1%, and 47.5%, respectively. According the SUCRA evaluation, SSNB was associated with the best effectiveness of relieving post-stroke hemiplegic shoulder pain, followed by intra-muscular BoNT injection, intra-bursal BoNT injection, intra-articular corticosteroid injection, intra-articular HA injection and the placebo treatment at the fourth week after intervention (Table 3).

### 3.5. Comparison of WMD for VAS Reduction (4th to 24th Weeks)

A forest plot of pairwise meta-analysis for the WMDs between the different injection therapies is presented in Figure 2B. For the network meta-analysis, the network graph is shown in Figure 3B, disclosing the geometry of the treatment network. The forest plot of network comparisons is presented in Figure 4B. The league tables for the pairwise and network meta-analyses are summarized in Table S2. The rank probability results and the values of SUCRA are demonstrated in Figure 5B and Table 3, respectively.

In the pairwise meta-analysis (Figure 2B), SSNB and intra-articular/bursal corticosteroid injections were significantly superior to the placebo, with WMDs of 1.75 (95% CI, 0.98 to 2.51) and 1.96 (95% CI, 1.10 to 2.81), respectively. SSNB was inferior to intramuscular BoNT injections (WMD, −1.85; 95% CI, −2.68 to −1.02). No significant differences were found in the following comparisons: intra-muscular BoNT or intra-articular HA injections vs. placebo; SSNB, intra-articular HA, intra-muscular BoNT, and intra-bursal BoNT injections vs. intra-articular/bursal corticosteroid injections.

In the network meta-analysis (Figure 4B), intramuscular BoNT injections appeared to be significantly better than the placebo, with a WMD of 1.57 (95% CI, 0.30 to 2.84). We did not identify significant differences among the other pair comparisons. In terms of the effect of pain relief between the 4th week and 24th week following intervention, the simulation of rank probabilities (Figure 5B) showed that intra-muscular BoNT injections had the highest probability (79.8%) as the best treatment, whereas intra-bursal BoNT injections tended to be the second-best therapy (the probability: 55.2%). Furthermore, intra-articular/bursal corticosteroid and intra-articular HA injections were likely to be the third- or fourth-best treatments, with probabilities of 48.5% and 34.7%, respectively. SSNB had the highest probability (65.4%) as the fifth-best treatment. Based on the SUCRA evaluation, intra-muscular BoNT injection was associated with the best effectiveness of relieving post-stroke hemiplegic shoulder pain, followed by intra-bursal BoNT injection, intra-articular corticosteroid injection, intra-articular HA injection, SSNB and the placebo treatment at between the 4th and 24th weeks after intervention (Table 3).

### 3.6. Sensitivity Analysis

A sensitivity analysis was performed by exclusion of the non-RCTs. The effect of intra-bursal BoNT injections was not examined in this network, because the only study [34] that used the aforementioned treatment was not a RCT. None of the effect sizes of the available network comparisons changed the direction of association after excluding the non-RCTs (Figure S4 and Table S3).

### 3.7. Publication Bias

No asymmetry of the intergroup comparisons regarding WMDs at the fourth-week or between the 4th and 24th weeks following intervention was recognized on the corresponding funnel plots (Figure S5). No significant small study bias was detected in the Egger’s test (*p* = 0.987 for the WMDs at the fourth-week and 0.909 for the WMDs between the 4th and 24th weeks).

## 4. Discussion

The network meta-analysis revealed that all the five injection therapies had higher probabilities of being better than the placebo at reducing HSP. At the fourth-week following the interventions, SSNB was likely to rank first, followed by intramuscular BoNT injections. Concerning the period between the 4th and 24th weeks, intramuscular BoNT injections appeared to be the most effective alternative for treating HSP.

Intra-bursal BoNT injections ranked second in relieving the symptoms of HSP between the 4th and 24th weeks. The main concern is that in our network meta-analysis, only one study [34] employed intra-bursal BoNT injections. Furthermore, the aforementioned study did not use a randomized controlled design. Therefore, the effect of intra-bursal BoNT injections could not be confirmed though the sensitivity analysis by excluding non-RCTs. Although a recent meta-analysis reported the superiority of intra-articular/bursal injections of BoNT over corticosteroids in the management of chronic shoulder pain between the first and third months after treatment [13], more evidence is still needed to validate the benefits of intra-bursal BoNT injections for treating HSP.

The best relief of HSP seemed to be provided by SSNB at the fourth post-injection week. According to our included studies, local anesthetics, including lidocaine [11,39,42] and bupivacaine [41,46], were the main regimens used for SSNB. The onset time ranged between two (lidocaine) and five (bupivacaine) minutes [47], enabling SSNB to take effect rapidly. Although the maximum effective duration ranges from one (lidocaine) to four (bupivacaine) hours [47], the clinical effect of SSNB seemed to persist in the fourth week in our analysis. The mechanism of extended symptom relief is not clear. We also observed that some of the included trials also added corticosteroids or physiological serum into the injectate [11,39,41,42], which might reduce neurogenic inflammation and potentiate the effective duration of SSNB [48].

Intramuscular BoNT injections ranked second in the treatment outcomes of HSP at the fourth post-intervention week. Spasticity, defined as a velocity-dependent increase in muscle tone [9], commonly involves the muscles over the shoulder girdle, e.g., subscapularis, teres major, pectoralis major, latissimus dorsi muscles after stroke [9]. Accordingly, the spasticity of such muscles frequently leads to painful/limited shoulder motions. BoNT is an exotoxin produced by *Clostridium botulinum*, and its intramuscular injection inhibits the release of acetylcholine at the neuromuscular junction [49]. The maximum effectiveness of intramuscular BoNT injections is usually seen in the second or third post-intervention week but may be delayed due to muscle fibrosis after prolonged paresis in patients that have had a stroke [50]. This issue might have accounted for its lower rank than SSNB in the fourth-week.

Nonetheless, between the fourth and twenty-fourth weeks, intramuscular BoNT injections were found to be the best treatment for HSP. This finding is consistent with our prior assumptions. First, spasticity accounts for the leading cause of persistent HSP. A reduction of spasticity facilitates the normalization of shoulder motion and the reduction of the associated pain. Second, the maximum effect of intramuscular BoNT injections had been achieved in most of our included studies one month after the injection. The active duration of BoNT is at least three months [51], further enabling patients that have had a stroke to benefit from sustained relief of spasticity-related pain.

Our meta-analysis revealed that the doses and target muscles for intra-muscular BoNT injections varied among the included studies. Although we pooled them together for the purpose of network comparisons, the grouped finding of pain control might not be attributed to the motor inhibition only. Aside from neuromuscular blockade, animal studies found that the administration of BoNT was associated with a reduction of substance P release as well as subsequent neurogenic inflammation [52,53]. More basic research is needed to investigate the mechanism of relieving HSP through intra-muscular administration of BoNT.

In our network meta-analysis, corticosteroid injections were consistently better than a placebo at both time points. Considering its well-established effects and thoroughly investigated adverse reactions in treating musculoskeletal pain [54], intra-articular/bursal corticosteroid injections can be considered as useful alternatives for HSP, especially in patients with concomitant rotator cuff or glenohumeral joint pathologies. Compared with corticosteroid injections, a previous meta-analysis revealed the non-superiority of intra-articular HA for shoulder pain management [55]. In our analysis, HA injections mostly ranked behind other non-placebo treatments, and as such, they cannot be recommended for the management of HSP.

Furthermore, SSNB was found to be the least effective among all the non-placebo treatments between the 4th and 24th weeks. This finding can be attributed to the fading effect of local anesthetics. Although SSNB is effective in pain relief by blocking sensory impulses, it does not treat the underlying causes of pain. On the other hand, intramuscular/bursal/articular administration of BoNT or corticosteroid intervenes potential pain generators (spasticity or rotator cuff pathologies).

In our meta-analysis, if the treatment was a combination of two therapies, and this combination was shown in only one arm of the included studies, we would discard this therapeutic arm from the pooled analysis due to its low representativeness [46]. Furthermore, a recent systematic review reported that addition of corticosteroid to local anesthetics had only a small or no effect on the improvement of chronic non-cancer pain compared with local anesthetics alone [56]. Therefore, if one study comprised two similar treatment arms (e.g., one group received SSNB with local anesthetic and the other group underwent the same block with additional corticosteroids) [42], we combined the data from both arms for the analysis.

Our network meta-analysis yielded several clinical implications. First, SSNB can be used as the first-line injection therapy in HSP due to its rapid onset. However, its effect is less sustaining, and repeat blocks or subsequent treatments targeting the underlying pain generators might be required. Second, if there is concomitant upper extremity spasticity, BoNT injections for the spastic periarticular muscles should be prioritized in managing HSP.

The current meta-analysis had several limitations. First, not all of the included studies were RCTs. Our analysis might be influenced by confounding, selection, information, and reporting biases [57]. However, we believed that the influence was minimal, as only two of the enrolled studies used a non-randomized controlled design. The concern could be partly resolved by the sensitivity analysis, which revealed no change of the direction of associations regarding the effect sizes from network comparisons after exclusion of the non-RCTs. Second, only a few of the included trials provided an assessment of shoulder function. Therefore, we were not able to analyze whether the functional improvement was consistent with pain reduction after the different injection therapies. Third, the loop inconsistency models showed some significant inconsistency between the direct and indirect comparison between the 4th and 24th weeks. This might be partially attributed to differences in the timing of data extraction across the retrieved studies. Therefore, further studies using standardized time frames to validate the effects of each injection at various post-stroke stages are required. Fourth, high heterogeneity was shown in the direct comparison between intra-muscular BoNT injections and placebo. We speculated that the heterogeneity was derived from significant variations of the doses of BoNT per target muscle across different trials. More studies are needed in the future for justifying the most suitable BoNT dosage during intra-muscular injections for relieving hemiplegic shoulder pain. Fifth, not all the included studies had enrolled a large number of patients. Different risks of bias could be identified though the quality assessment, which interfered the estimation of true effectiveness. Furthermore, as the ranking of the treatment effects in a network meta-analysis is derived from simulation, interpretation should be cautious and more high-quality trials are still required to justify the preliminary observations.

## 5. Conclusions

The current systematic review and meta-analysis found that SSNB was likely to rank first in relieving HSP at the fourth post-treatment week although the probability of being the best treatment was approximately 50%. Furthermore, care should be taken due to its short duration of effectiveness and a lack of enough studies with head-to-head comparisons of SSNB vs. other injection regimens. Intra-muscular BoNT injections seem to be the best treatment in the post-injection period between the 4th and 24th weeks. Concomitant spasticity in the shoulder girdle muscles should be evaluated as a potential source of HSP and properly managed using intra-muscular BoNT injections. If rotator cuff pathologies are suspected clinically, intra-articular/bursal corticosteroid injections can be administered. Further prospective studies are warranted to investigate the combined efficacy of different injections and their long-term therapeutic efficacy in treating HSP.

## Figures and Tables

**Table 1:** Summary of the retrieved trials investigating the injection therapies for hemiplegic shoulder pain due to stroke.

Author, Year	Trial Design	Blinding	Allocation Concealment	Inclusion Criteria	Patient Characteristics	Intervention Arm	Case Number (Male/Female)	Age (Year)	Post-Stroke Follow-Up (Months) *
Kasapoğlu-Aksoy et al., 2020	RCT	Double-blinded (lack of details)	Not mentioned	Pain ≥ 3 weeks with VAS ≥ 4; MAS 3–4 with abduction and external rotation limitation	Stroke onset for more than 6 months	IM BoNT	30 (19/11)	58.47 ± 14.68 ^1^	11 (6 to 34) ^4^
						SSNB	27 (16/11)	59.89 ± 10.57 ^1^	10 (6 to 28) ^4^
Terlemez et al., 2020	RCT	Double-blinded (patients and assessors)	Yes	VAS of pain > 3	Stroke onset within 24 months	SSNB (local anesthetics + corticosteroid)	10 (7/3)	60.0 (58.0 to 75.0) ^3^	13.0 (11.0 to 15.0) ^3^
						SSNB (local anesthetics)	10 (4/6)	64.0 (52.0 to 65.0) ^3^	14.5 (12.0 to 24.0) ^3^
						Placebo	10 (4/6)	57.5 (56.0 to 66.0) _3_	15.0 (12.0 to 18.0) ^3^
Aydin et al., 2019	Quasi-experimental trial	No blinding	No	Pain ≥ 3 months	Stroke with resultant hemiplegia	SSNB	21 (8/13)	65.1 ± 8.8 ^1^	4.4 ± 1.7 ^1^
						Control	20 (10/10)	62.7 ± 10.5 ^1^	5.2 ± 2.0 ^1^
Sencan et al., 2019	RCT	Double-blinded (patients and assessors)	Not mentioned	VAS of pain ≥ 4	Stroke onset within 12 months	IA corticosteroid	10 (6/4)	61.4 ± 6.3 ^1^	5.8 ± 2.0 ^1^
						SSNB	10 (5/5)	64.5 ± 8.6 ^1^	5.3 ± 1.4 ^1^
						IA corticosteroid + SSNB	10 (6/4)	62.9 ± 9.8 ^1^	5.4 ± 2.1 ^1^
Wu et al., 2019	Retrospective cohort study	No blinding	Not mentioned	Pain ≥ 2 months, VAS of pain > 3 at rest or >5 at shoulder abduction; MAS < 2; sonographic diagnosed rotator cuff disorder or bursitis	Stroke with resultant hemiplegia	IB BoNT	18 (10/8)	61.4 ± 13.0 ^1^	6.3 ± 4.7 ^1^
						IB corticosteroid	20 (11/9)	66.2 ± 9.8 ^1^	4.9 ± 5.6 ^1^
Huang et al., 2018	RCT	Double-blinded (patients and assessors)	Yes	VAS of pain ≥ 3	Stroke onset within 6 months	Hyaluronic acid	18 (11/7)	59.7 (10.6) ^2^	3.0 (1.3) ^2^
						Placebo	9 (6/3)	62.0 (9.3) ^2^	2.9 (2.4) ^2^
Jang et al., 2016	RCT	Single-blinded (patients)	Yes	Pain WBS score ≥ 2; passive ROM limitation of a capsular pattern	Stroke onset within 3 months	Hyaluronic acid	21 (13/8)	56.6 ± 11.3 ^1^	1.9 ± 1.0 ^1^
						IA corticosteroid	18 (14/4)	60.8 ± 13.7 ^1^	1.8 ± 1.1 ^1^
Adey-Wakeling et al., 2013	RCT	Double-blinded (patients and assessors)	Yes	VAS of pain ≥ 3	Stroke onset within 12 months	SSNB	32 (21/11)	0 to 65 y/o: n = 15 66 to 79 y/o: n = 9 ≥80 y/o: n = 8	3.3 ± 2.3 ^1^
						Placebo	32 (15/17)	0 to 65 y/o: n = 16 66 to 79 y/o: n = 13 ≥80 y/o: n = 3	2.8 ± 2 ^1^
Marciniak et al., 2012	RCT	Double-blinded (patients and assessors)	Yes	VAS of pain ≥ 4; MAS ≥ 3 in shoulder adductor or internal rotator	Stroke with resultant hemiplegia or hemiparesis	IM BoNT	10 (6/4)	60.2 ± 7.8 ^1^	28.8 ± 38.5 ^1^
						Placebo	11 (7/4)	59.8 ± 10.3 ^1^	46.5 ± 84.5 ^1^
Rah et al., 2012	RCT	Triple-blinded (patients, physicians and assessors)	Yes	Pain ≥ 1 month and VAS ≥ 3; clinically diagnosed rotator cuff disorder; deltoid muscle power ≥ 2; MMSE ≥ 20	Stroke with resultant hemiplegia	IB corticosteroid	29 (21/8)	56.6 ± 12.5 ^1^	23.6 ± 16.9 ^1^
						Placebo	29 (18/11)	54.9 ± 10.6 ^1^	18.8 ± 10.7 ^1^
Lakse et al., 2009	RCT	Not blinded	Not mentioned	Pain caused by frozen shoulder or subacromial impingement syndrome	Stroke more than 8 weeks	IA or IB corticosteroid	21 (10/11)	62.2 ± 9.1 ^1^	10 (3 to 22) ^4^
						Placebo	17 (8/9)	66.3 ± 6.7 ^1^	7 (2 to 64) ^4^
De Boer et al., 2008	RCT	Double-blinded (lack of details)	Not mentioned	Pain ≥ 1 week and VAS ≥ 4; MAS ≥ 1; passive external rotation limitation of the humerus ≥ 50% compared with the unaffected side	Stroke with spastic hemiplegia	IM BoNT	10 (6/4)	58.5 ± 10.3 ^1^	9.3 (17.1) ^2^
						Placebo	11 (6/5)	56.3 ± 7.6 ^1^	4.9 (5.3) ^2^
Lim et al., 2008	RCT	Double-blinded (patients and assessors)	Yes	Pain ≤ 12 weeks and VAS of pain ≥ 6; passive external rotation limitation ≥ 20°	Stroke within 24 months	IM BoNT	16 (8/8)	64.8 ± 2.1 ^1^	7.7 ± 1.8 ^1^
						IA corticosteroid	13 (7/6)	57.1 ± 3.6 ^1^	10.0 ± 2.5 ^1^
Kong et al., 2007	RCT	Double-blinded (patients and assessors)	Yes	Pain ≥ 2 weeks and VAS of pain ≥ 4; MAS ≥ 2 in shoulder adductor and elbow flexor	Stroke for more than 3 months	IM BoNT	7 (3/4)	46.3 ± 9.0 ^1^	8.3 ± 7.0 ^1^
						Placebo	9 (8/1)	56.0 ± 13.6 ^1^	10.1 ± 6.5 ^1^
Marco et al., 2007	RCT	Double-blinded (patients and assessors)	Yes	Pain ≥ 3 months and VAS of pain ≥ 4; MAS ≥ 3	Stroke for more than 3 months	IM BoNT	14 (10/4)	63.9 ± 10.6 ^1^	5.8 (3.0 to 8.8) ^3^
						Placebo	15 (11/4)	67.2 ± 7.4 ^1^	4.4 (3.7 to 7.0) ^3^
Yelnik et al., 2007	RCT	Double-blinded (lack of details)	Yes	MAS ≥ 1+ in medial rotator or elbow flexor; passive external rotation limitation 10° or <30° compared to the opposite side	Stroke regardless of the stage	IM BoNT	10 (7/3)	53.0 ± 4.6 ^1^	7.5 ± 6.2 ^1^
						Placebo	10 (8/2)	55.2 ± 8.3 ^1^	26.5 ± 35.0 ^1^
Snels et al., 2000	RCT	Double-blinded (patients and assessors)	Yes	Pain ≥ 2 weeks and VAS of pain ≥ 4; passive external rotation limitation > 20°	Stroke with resultant hemiplegia	IA corticosteroid	18 (12/6)	60.6 ± 8.4 ^1^	<6 months: n = 12 ≥6 months: n = 6
						Placebo	19 (7/12)	62.5 ± 10.6 ^1^	<6 months: n = 14 ≥6 months: n = 5

Data format: ^1^ indicates mean ± standard deviation. ^2^ indicates median (interquartile range). ^3^ indicates median (25 percentile value to 75 percentile value). ^4^ indicates median (minimum value to maximum value). * Post-stroke follow-up were all converted to months by dividing the days with 30 and multiplying the years with 12, and all the converted data were rounding to the first decimal place. If the data were originally expressed in months, they were remained unchanged. Abbreviations: BoNT, botulinum toxin; IA, intra-articular; IB, intra-bursal; IM, intra-muscular; MAS, modified Ashworth scale; MMSE, mini-mental state examination; RCT, randomized control trial; ROM, range of motion; SSNB, suprascapular nerve block; VAS, visual analog scale; WBS, Wong-Baker scale; y/o, year-old.

**Table 2:** Summary of intervention details in the retrieved trials.

Author, Year	Trial Arm	Intervention Detail	Guidance	Inclusion for Meta-Analysis	Outcome for Meta-Analysis	Secondary Outcomes	Follow-Up (Week)
Kasapoğlu-Aksoy et al., 2020	IM BoNT	Total 140 to 210 units BoNT (Botox) per person, 100 to 150 units into pectoralis major muscle and 40 to 60 units into teres major muscle	Ultrasound	Included	VAS (at rest)	ROM, FMS, MAS	2, 6
	SSNB	1 mL triamcinolone + 9 mL 2% lidocaine at suprascapular notch	Ultrasound	Included			
Terlemez et al., 2020	SSNB (local analgesic + steroid)	5 mL 2% lidocaine + 1 mL betamethasone at the suprascapular notch	Ultrasound	Included with data combination	VAS (during motion)	ROM	1, 4
	SSNB (local analgesic)	5 mL 2% lidocaine at the suprascapular notch	Ultrasound				
	Placebo	5 mL 2% lidocaine injected into trapezius muscles	Ultrasound	Included			
Aydin et al., 2019	SSNB	1 mL betamethasone + 2 mL 10% lidocaine + 2 mL physiologic serum at the suprascapular fossa	Ultrasound	Included	VAS (during motion)	ROM, MAS, Brunnstrom stage, EQ-5 D-3 L	1, 4, 12
	Control	Passive and active-assistive ROM exercises (3 sets daily, 20 times in each set)	Not available	Included			
Sencan et al., 2019	IA corticosteroid	40 mL methylprednisolone + 1 mL 0.5% bupivacaine + 2 mL saline into the glenohumeral joint	Fluoroscopy	Included	VAS (during motion)	ROM, MAS, MBI	2, 8
	SSNB	3 mL 0.5% bupivacaine + 2 mL saline at suprascapular notch	Fluoroscopy	Included			
	IA corticosteroid + SSNB	Combination of aforementioned two treatments	Fluoroscopy	Excluded			
Wu et al., 2019	IB BoNT	100 units BoNT (Botox) into the subacromial-subdeltoid bursa	Ultrasound	Included	VAS (at rest)	FMS	2, 4, 8, 12
	IB corticosteroid	1 mL betamethasone + 2 mL 2% lidocaine + 1 mL saline into the subacromial-subdeltoid bursa	Ultrasound	Included			
Huang et al., 2018	Hyaluronic acid	2.5 mL sodium hyaluronate (ARTZ Dispo) into the subdeltoid bursa; total 3 doses (1 dose per week)	Ultrasound	Included	VAS (at rest)	ROM, MAS, FMS, shoulder subluxation, soft tissue hyperemia	4, 12
	Placebo	2.5 mL saline into the subdeltoid bursa	Ultrasound	Included			
Jang et al., 2016	Hyaluronic acid	2 mL high molecular weight sodium hyaluronate + 4 mL 0.5% lidocaine (total 3 doses in a week)	Ultrasound	Included	Pain rating scale of WBS (0–10)	ROM	1, 4, 8
	IA corticosteroid	40 mg triamcinolone + 4 mL 0.5% lidocaine + 1 mL saline into the shoulder joint	Ultrasound	Included			
Adey-Wakeling et al., 2013	SSNB	40 mg methylprednisolone + 0.5% 10 mL bupivacaine into the supraspinatus fossa	Landmark	Included	VAS (not specified)	MRS, Croft Disability Index, EuroQol Health Questionnaire	1, 4, 12
	Placebo	5 mL normal saline subcutaneously to the same region of the shoulder	Landmark	Included			
Marciniak et al., 2012	IM BoNT	Total 140 to 200 units BoNT (Botox) per person, with 100 to 150 units into pectoralis major muscles and 40 to 60 units into teres major muscles if shoulder extensors MAS ≥ 3	Electromyography	Included	VAS (daily worst pain)	ROM, FMS, MAS, MPQ, DAS, Beck depression inventory	2, 4, 12
	Placebo	2 mL of saline into pectoralis major and teres major muscles	Electromyography	Included			
Rah et al., 2012	IB corticosteroid	40 mg triamcinolone + 1 mL 1% lidocaine into the subdeltoid bursa	Ultrasound	Included	VAS (at night)	ROM, MBI, SDQ	2, 4, 8
	Placebo	5 mL 1% lidocaine into the subdeltoid bursa	Ultrasound	Included			
Lakse et al., 2009	IA or IB corticosteroid	1 mL triamcinolone + 9 mL prilocaine into the shoulder joint in frozen shoulders, or into the subacromial bursa in impingement syndrome	Landmark	Included	VAS (at rest)	ROM, MAS, BI, Brunnstrom stage	1, 4
	Placebo	Not mentioned	Landmark	Included			
De Boer et al., 2008	IM BoNT	Total 50 units BoNT (Botox) into subscapularis muscle	Landmark	Included	VAS (not specified)	ROM	0, 6, 12
	Placebo	1 mL saline into subscapularis muscle	Landmark	Included			
Lim et al., 2008	IM-BoNT	BoNT (Botox) into infraspinatus, subscapularis or pectoralis muscles; maximal dose: 50 units in each muscle and 100 units in each patient	Landmark	Included	NRS (during motion)	ROM, FMS, MAS Physician’s global rating scale	2, 6, 12
	IA corticosteroid	40 mg triamcinolone in shoulder joints	Landmark	Included			
Kong et al., 2007	IM-BoNT	250 units BoNT (Dysport) to pectoralis major muscles and 250 units BoNT (Dysport) to biceps brachii muscles	Landmark	Included	VAS (not specified)	ROM, MAS	4, 8, 12
	Placebo	2.5 mL saline into pectoralis major and biceps brachii muscles	Landmark	Included			
Marco et al., 2007	IM BoNT	500 units BoNT (Dysport) into pectoralis major muscles	Electromyography	Included	VAS (during motion)	ROM, MAS	1, 4, 12, 24
	Placebo	2.5 mL of saline into pectoralis major muscles	Electromyography	Included			
Yelnik et al., 2007	IM BoNT	500 units BoNT (Dysport) into subscapularis muscles	Electromyography	Included	VAS (not specified)	ROM, MAS	1, 2, 4
	Placebo	Solvent (for BoNT) into subscapularis muscle	Electromyography	Included			
Snels et al., 2000	IA corticosteroid	40 mg triamcinolone into shoulder joints; total 3 doses (0, 1st, 3rd week)	Landmark	Included	VAS (not specified)	ROM, FMS, BI, Action Research Arm test, Rehabilitation Activities Profiles	6, 12
	Placebo	1 mL saline into shoulder joints, total 3 doses (0, 1st, 3rd week)	Landmark	Included			

Abbreviations: BI, Barthel index; BoNT, botulinum toxin; DAS, disability assessment scale; EQ-5D-3L, three-level of EuroQol five-dimensional; FMS, Fugl-Meyer scale; IA, intra-articular; IB, intra-bursal; IM, intra-muscular; MAS, modified Ashworth scale; MBI, modified Barthel index; mg, milligram; mL, milliliter; MPQ, McGill pain questionnaire; MRS, Modified Rankin Scale; NRS, numeric rating scale; ROM, range of motion; SDQ, shoulder disability questionnaire; SSNB, suprascapular nerve block; VAS, visual analog scale; WBS, Wong-Baker scale.

**Table 3:** Surface Under the Cumulative Ranking (SUCRA) of the reduction of the visual analogue scale (VAS) at the 4th week and between the 4th and 24th weeks.

	VAS Reduction at the 4th Week		VAS Reduction between the 4th and 24th Weeks	
Rank	Treatment	SUCRA	Treatment	SUCRA
1	SSNB	88.6	IMBoNT	93.9
2	IMBoNT	81.4	IBBoNT	74.5
3	IBBoNT	52.7	Steroid	52.4
4	Steroid	37.7	HA	49.1
5	HA	37.2	SSNB	30.0
6	Placebo	1.4	Placebo	0.1

Abbreviation: BoNT, botulinum toxin; HA, hyaluronic acid; IB, intra-bursal; IM, intra-muscular; SSNB, suprascapular nerve block.

## Data Availability

Data is contained within the article and supplementary material.

## Supplementary Materials

The following are available online at https://www.mdpi.com/article/10.3390/ph14080788/s1, Table S1: League table for the reduction of the visual analogue scale of pain at the 4th week; Table S2: League table for the reduction of the visual analogue scale of pain between the 4th and 24th week; Table S3: Network comparisons of the treatment effects before and after exclusion of the non-randomized controlled trials; Figure S1: Risk of bias assessment of the included studies; Figure S2: Risk of bias graph of the included studies; Figure S3: Loop inconsistency plots for different injection therapies in terms of VAS reduction; Figure S4: Forest plots of the network estimates following exclusion of the non-randomized controlled trials; Figure S5: Funnel plots for the effect sizes between different injection therapies in terms of the VAS reduction.

## Abbreviations and Acronyms

BoNT	Botulinum toxin
HA	Hyaluronic acid
HSP	Hemiplegic shoulder pain
RCT	Randomized controlled trial
SSNB	Suprascapular nerve block
VAS	Visual analog scale
WMD	Weight mean difference
95% CI	95% confidence interval
